# Gastric juice piR‐1245: A promising prognostic biomarker for gastric cancer

**DOI:** 10.1002/jcla.23131

**Published:** 2019-11-28

**Authors:** Xiaorong Zhou, Jianhong Liu, Aifeng Meng, Lihong Zhang, Min Wang, Hong Fan, Wei Peng, Jianwei Lu

**Affiliations:** ^1^ Department of Medical Oncology Jiangsu Cancer Hospital Jiangsu Institute of Cancer Research Nanjing Medical University Affiliated Cancer Hospital Nanjing China; ^2^ Department of Gastroenterology First People's Hospital of Yunnan Province Kunming China

**Keywords:** biomarker, gastric cancer, gastric juice, piR‐1245, PIWI‐interacting RNA

## Abstract

**Background:**

Emerging reports demonstrated that PIWI‐interacting RNAs (piRNAs) played an indispensable role in tumorigenesis. However, it still remains elusive whether piR‐1245 in gastric juice specific in stomach could be employed as a biomarker for gastric cancer (GC). The present work is aiming at exploring the possibility of piR‐1245 in gastric juice as a potential marker to judge for diagnosis and prognosis of gastric cancer.

**Methods:**

Gastric juice was collected from 66 GC patients and 66 healthy individuals. Quantitative real‐time reverse transcriptase polymerase chain reaction (qRT‐PCR) was employed to measure the levels of piR‐1245 expression. Then, the pattern of piR‐1245 expression in gastric juice was determined between GC patients and healthy individuals. A receiver operating characteristic (ROC) curve was constructed for distinguishing GC from healthy individuals.

**Results:**

Gastric juice piR‐1245 levels in GC were higher than those of controls (*P* < .0001). The value of area under ROC (AUC) was 0.885 (sensitivity, 90.9%; specificity, 74.2%; 95% confidence interval, 0.8286 to 0.9414). High gastric juice piR‐1245 expression was signally correlated with tumor size (*P* = .013) and TNM stage (*P* = .001). GC patients with high piR‐1245 expression in gastric juice exerted a poorer overall survival (OS) (*P* = .0152) and progression‐free survival (PFS) (*P* = .013). COX regression analysis verified that gastric juice piR‐1245 expression was an independent prognostic risk variable for OS (*P* < .05).

**Conclusions:**

The current study suggested that piR‐1245 in gastric juice had the potential to be a useful biomarker for GC detection and prognosis prediction.

## 1 INTRODUCTION

Gastric cancer (GC) is a frequent malignancy all over the world, and its incidence has been increasing evidently over the past decade.^1^ A large proportion of GC patients are diagnosed at the middle and late stages, due to no specific symptoms and early effective screening methods in GC.^2^ Gastroscope is helpful for GC detection, but its invasive discomfort limits its wide application. Meanwhile, carcinoembryonic antigen (CEA) and carbohydrate antigen 724 (CA724) is short of satisfactory specificity and sensitivity in GC diagnosis.^3^, ^4^, ^5^ Hence, there is an urgent need for accurate and reliable biomarkers to advance diagnosis and prognosis for GC.

Piwi‐interacting RNAs (piRNAs) are a new type of non‐coding RNAs, which are of interest mainly because of their important role in maintaining germ cells.^6^ Latest reports demonstrated piRNAs were widely found in mammalian somatic cells especially in the human cancers.^7^ Accumulating literatures verified that piRNAs are deregulated in a variety of tumor tissues.^8^ Aberrant expression of piRNA is a key character with feasible implications for diagnosis or prognosis in multiple types of cancers.^9^ Besides, gastric juice (GJ) can provide useful information and has diagnostic value in GC.^10^, ^11^, ^12^ Prominently, piRNAs have been disclosed to be present in a stable form in human body fluids, including the plasma and serum.^13^, ^14^, ^15^, ^16^ Moreover, Qu et al suggested that serum piRNAs expression could be used to be biomarkers for CRC detection and to predict prognosis.^14^ Based on these studies, we deduced that detection of piRNAs in GJ might provide a non‐invasive tool for diagnosis and prognosis for GC. However, the potential clinical value of GJ piRNAs in GC still remains to be elusive until now.

Here, we concentrate on the piR‐1245. Previous report demonstrated that high expression of piR‐1245 was associated with progression of colorectal cancer (CRC).^17^ Functional studies elucidated that it boosted CRC cell proliferation and contributed to tumor metastasis.^17^ Moreover, piR‐1245 could be served as a novel oncogene and a feasible prognostic biomarker for CRC.^17^ These results suggested that piR‐1245 could show an important role in CRC tumorigenesis. However, the role of piR‐1245 in GC remains largely elusive.

In present study, we firstly explored the expression pattern of piR‐1245 in GJ; then, we uncovered its clinical value in the GJ as an additional indicator for GC. Moreover, the relationship between the levels of GJ piR‐1245 expression and the survival rate of GC patients was identified to elucidate its possibility for prognostic prediction.

## 2 MATERIALS AND METHODS

### 2.1 Patients

Gastric juice samples were collected from 66 patients and 66 normal persons in the First People's Hospital of Yunnan Province (Kunming, China). All diagnoses were reviewed and by pathologic diagnosis of biopsies via gastroscopy. Histological samples from all individuals were confirmed to GC diagnosis of, and the tumors were staged referring to the 8th edition of the AJCC TNM staging systems. The normal persons were reviewed to confirm that they had no history of cancer. The clinicopathological characteristics of GC patients were retrospectively collected.

### 2.2 Sample preparation

Briefly, 5 mL of GJ specimens were collected from patients and healthy controls. GJ was harvested within 1 hour of its collection by sequential centrifugation (2500 rpm for 20 minutes at 4°C, 12 000 rpm for 30 minutes at 4°C) to completely dislodge impurity. Then, all specimens were transferred into RNase‐free tubes and stored in liquid nitrogen for further research. To avoid piRNA pollution from other sources, the exclusion criteria included benign gastric disease and other gastrointestinal cancer, for example, e.g. bile reflux gastritis, upper gastroduodenal bleeding, oral and esophageal cancer, and the coexistence of other malignant diseases in this study.^18^

### 2.3 RNA extraction

Total RNA Extraction was collected according to the manufacturer's specification. In brief, 250 ul GJ was fully mixed with 750 uL TRIzol Reagent (Invitrogen). Then, 200 uL of chloroform was added. The mixture was thoroughly incorporated and centrifuged at 12 000 rpm at 4°C for 30 minutes. Then RNA concentration and purity were determined using NanoDrop ND‐1000.^19^

### 2.4 Quantitative real‐time reverse transcriptase polymerase chain reaction *(qRT‐PCR)*

Reverse transcription was implemented following the manufacturer's guidance. Quantitative polymerase chain reaction (qPCR) analysis was performed using the First‐Strand cDNA Synthesis Kit (Takara) on Applied Biosystems 7900HT Real­Time PCR System (Applied Biosystems, Inc) and was carried out as previously reported.^20^ The primer sequences referred to previous literature.^21^ The method was employed for analysis as described previously.^17^

### 2.5 Statistical analysis

Continuous variables were presented as the mean ± SD Statistical analyses were implemented using SPSS version 19.0 (IBM) and GraphPad Prism 5 (GraphPad Software Inc).^22^ Differences between the groups were statistically assessed using Student's *t* test. Clinical characteristics were tested using Pearson's chi‐squared test. Overall survival (OS) was measured by the time interval from first confirmation to cancer‐related death. Progression‐free survival (PFS) was determined by the time interval from the treatment to the first radiologic progression. Survival was estimated by Kaplan‐Meier analysis, and the differences were verified using the log‐rank test. COX regression analysis was employed for the univariate and multivariate analysis of risk factors. *P* < .05 were identified as statistically significant.

## 3 RESULTS

### 3.1 Determination of piRNA‐1245 levels in GJ and its value in GC diagnosis

**Figure 1:**
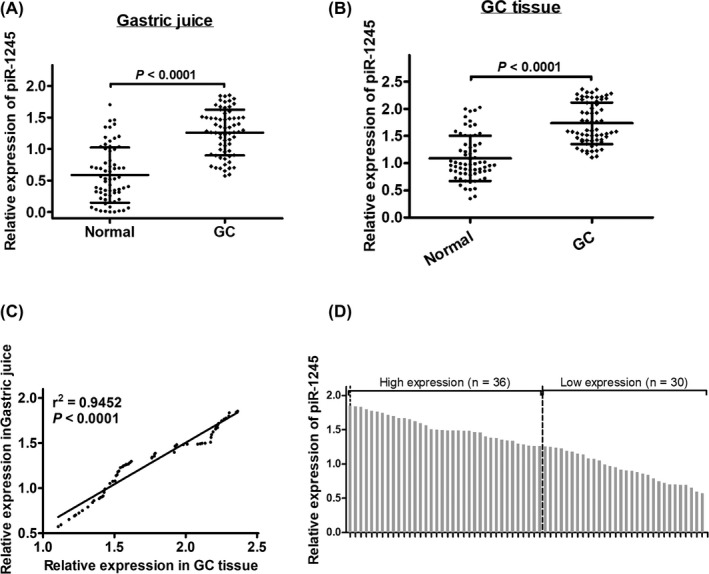
piR‐1245 expression levels in gastric fluid from GC patients and healthy controls. A & B, piR‐1245 expression levels in gastric fluid and tissues were significantly upregulated in GC patients compared with healthy controls (*P* < .0001). Data are shown as the mean ± standard deviation of experiments. C, Expression levels of piR‐1245 in gastric fluid were positively correlated with those in tissues in 66 human GC patients (*P* < .0001). D, piR‐1245 expression was classified into two groups

To determine the effect of piRNA‐1245 in GC, we firstly detected the levels of piRNA expression in GJ by qRT‐PCR. As can be seen from Figure 1A,B, the levels of piRNA‐1245 expression in GC patients' GJ and tissue were significantly higher than healthy normal (*P* < .0001). The relationship of piR‐1245 expression in GC patients' juice and tissues is very closely positively correlated (*r* ^2^ = .9452, *P* < .0001) (Figure 1C).

**Figure 2:**
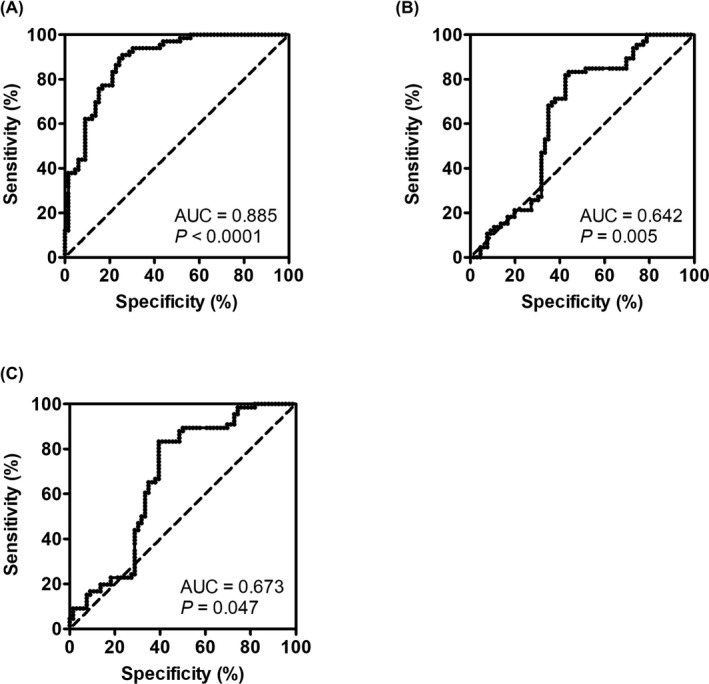
Receiver operating characteristic (ROC) analysis determining the feasibility of the levels of piR‐1245, CEA, and CA724 in gastric fluid to distinguish between GC patients and healthy individuals

To detect whether the pattern of piRNA‐1245 expression could be employed as a diagnostic indicator for GC, we established a ROC curve to compare with the different patterns of piRNA‐1245 expression in GC patients' GJ and controls. The value of cutoff point was 0.652. Area under the ROC curve (AUC) was 0.8850 (sensitivity, 90.9%; specificity, 74.2%; 95% confidence interval, 0.8286 to 0.9414; Figure 2A; Table 1). If CEA was used as the diagnostic marker of GC, AUC was 0.642 (sensitivity, 81.8%; specificity, 57.6%; 95% confidence interval, 0.5442 to 0.7405; *P* = .005; Figure 2B; Table 1). Moreover, if CA724 was employed as the biomarker for GC, AUC was 0.673 (sensitivity, 65.4%; specificity, 62.1%; 95% confidence interval, 0.5782 to 0.7658; *P* = .047; Figure 2C; Table 1). Therefore, the diagnostic value of piR‐1254 was better than that of CEA and CA724.

**Table 1:** Diagnostic efficiency of using piR‐1245, CEA or CA724 as a biomarker

Parameter	AUC	Sensitivity	Specificity	95% CI	*P*
piR‐1245	0.885	0.909	0.742	0.8286‐0.9414	<.0001*
CEA	0.642	0.818	0.576	0.5442‐0.7405	.005*
CA724	0.673	0.654	0.621	0.5782‐0.7658	.047*

* *P* < .05.

All results of this study showed that GJ piR‐1245 had good diagnostic value. Most important of all, it was non‐invasive in practice.

### 3.2 Relationship between piRNA‐1245 expression and clinicopathologic characteristics in GC patients

On the basis of the average ratio of relative piRNA‐1245 expression (1.26‐fold) in GJ of GC patients, 66 GC patients were divided into two groups: high group (n = 36, piRNA‐1245 expression ratio more than 1.26‐fold) and low group (n = 30, piRNA‐1245 expression ratio less than 1.26‐fold; Figure 1D). We examined that GJ piRNA‐1245 levels were related significantly with tumor size (*P* = .013) and TNM stage (*P* = .001; Table 2). However, we showed no significant correlation between piRNA‐1245 levels and gender, age, CEA, CA724, and histological grade (Table 2).

**Table 2:** Relationship of piR‐1245 with clinical characteristics of GC patients

Characteristic	n	piR‐1245 expression		*P*
		low	high	
Gender				
Male	36	14	22	.322
Female	30	16	14	
Age (years)				
＜60	29	12	17	.623
≥60	37	18	19	
Tumor size (cm)				
＜3.5	32	20	12	.013*
≥3.5	34	10	24	
TNM stage				
I‐II	21	16	5	.001*
III‐IV	45	14	31	
CEA (ng/mL)				
＜5	25	14	11	.210
≥5	41	16	25	
CA724 (U/mL)				
＜8.2	29	14	15	.804
≥8.2	37	16	21	
Histological grade				
Well‐moderate	28	15	13	.320
Poor‐signet	38	15	23	

Abbreviations: CA724, Carbohydrate antigen 724; CEA, Carcinoembryogenic antigen.

* *P* < .05.

### 3.3 Correlation of piRNA‐1245 expression with survival in GC patients

**Figure 3:**
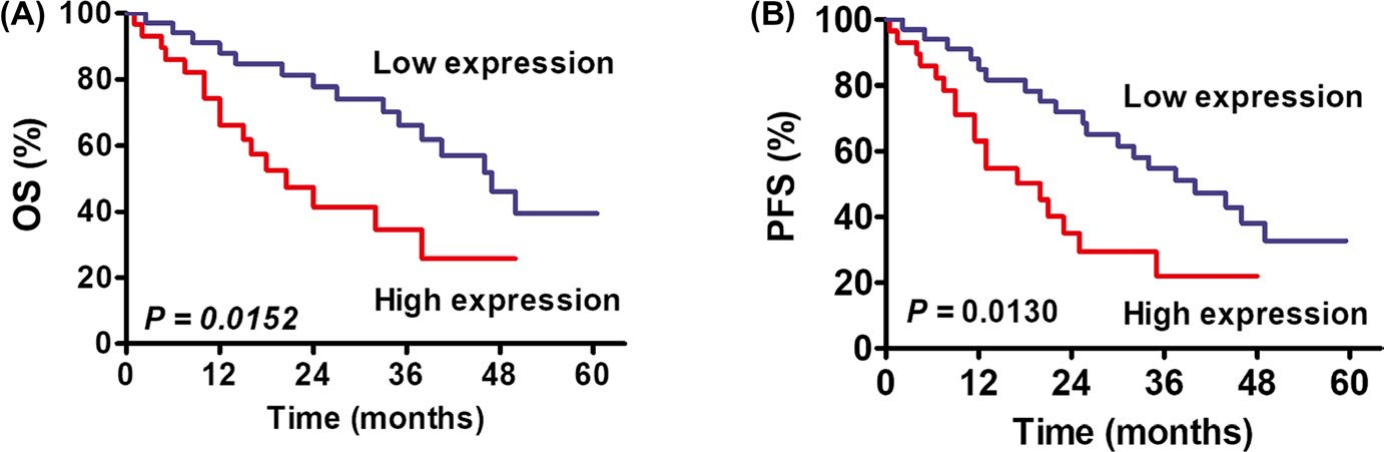
Survival analysis of patients with GC with high or low piR‐1245 expression in the gastric fluid. A, GC patients with a high piR‐1245 level had a shorter overall survival (OS) rate (*P* = .0152). B, GC patients with high piR‐1245 expression showed a longer progression‐free survival (PFS) time (*P* = .013)

We assessed the association of piRNA‐1245 with patients' survival. A total of 66 patients with complete medical records and adequate follow‐up were included in the analysis. Kaplan–Meier survival analysis exhibited that patients with high expression of piR‐1245 had significantly poorer OS and PFS rate compared with those with low expression of piR‐1245 (*P* = .0152 and *P* = .013, respectively; Figure 3). COX proportional hazards model proved that piR‐1245 expression was an independent prognostic trait for OS in GC patients (*P* = .041; Table 3).

**Table 3:** Univariate and multivariate analysis for overall survival

Variables	Univariate analysis			Multivariate analysis		
	HR	95% CI	*P*	HR	95% CI	*P*
Gender (Male vs Female)	1.671	0.729‐3.331	.069			
Age (< 60 vs ≥60)	3.259	2.168‐5.181	.083			
Tumor size (<3.5 vs ≥3.5)	1.349	0.875‐2.438	.016*	2.548	1.259‐4.015	.057
TNM stage (I‐II vs III‐IV)	1.284	0.670‐2.303	.038*	1.944	1.013‐3.887	.062
CEA (<5 vs ≥5)	2.195	0.778‐4.845	.075			
CA724 (<8.2 vs ≥8.2)	2.813	0.361‐5.019	.081			
Histological grade (Well‐moderate vs Poor‐signet)	2.345	1.294‐4.199	.068			
piR‐1245 expression (Low vs High)	2.183	0.996‐4.447	.027*	2.989	1.312‐6.393	.041*

Abbreviations: 95% CI, 95% confidence interval; HR, hazard ratio.

* *P* < .05.

## 4 DISCUSSION

Accumulating literatures showed that alternation of non‐coding RNAs in tissues and body fluids could be used as potential biomarkers for detection.^13^, ^14^, ^23^ Nevertheless, surprisingly little attention has been devoted to GJ piRNAs for GC up to now.

In this study, we attempted to use piRNAs in GJ as markers to distinguish gastric cancer. The detection of piRNA‐1245 in GJ could be a new avenue for differentiating GC from healthy people.

Increasing evidence uncovered non‐coding RNAs (ncRNAs), including microRNAs (miRNAs), long non‐coding RNAs (lncRNAs), circular RNAs (circRNAs), and transfer RNAs (tRNAs), could be used in GC diagnosis and prognosis.^24^, ^25^, ^26^, ^27^, ^28^, ^29^ Some altered ncRNAs played a crucial role in the regulation of malignant phenotypes of tumor.^24^ Mo et al elucidated that lncRNA RP11‐555H23.1 showed an important role in the occurrence of GC, and used as a new marker of GC.^26^ Zhu et al^29^ identified that tiRNA‐5034‐GluTTC‐2 might be responsible for a marker for GC diagnosis. Tan et al established that detection of miR‐145 and miR‐185 might be helpful for predicting adverse reactions and effectiveness of SOX regimen against GC.^27^ Recent reports illustrated that circ_0067582, circ_0005758, circ_0000467 and circ_0000181 could suggest a role in GC carcinogenesis, and be potential indicators in screening GC.^24^, ^25^, ^28^ Although accumulating ncRNAs have been identified, the diagnostic values of piRNAs still remains largely elusive.

The piRNAs were originally discovered in the Drosophila male germline.^18^ Recent reports demonstrated that piRNAs were of great importance for carcinogenesis.^7^, ^9^ It was also active in various cancer cells, implying that somatic cancer stem cells might adopt an unlimited proliferation potency via piRNA alteration.^30^ Zhang et al showed that piR‐651 was responsible for tumorigenesis of non‐small cell lung cancer by inhibiting its malignant behavior.^31^ Fu et al demonstrated that piRNA‐021285 played a crucial role in regulating methylome of breast cancer.^32^ Weng reported that piR‐1245 acted as an oncogene and promoted CRC progression.^21^ All of these are consistent with our observation. Our findings provide a viable source for non‐invasive diagnosis and prognosis of GC.

Because piRNAs are mostly only 26‐31 nucleotide (nt) in length, they are difficult to be degraded and own good stability. More importantly, they are easy to cross cell membrane. The latest report elucidated that plasma piRNAs were dramatically elevated in CRC patients comparing with healthy controls.^14^ Therefore, piRNAs are very suitable for non‐invasive biomarker of malignant diseases.

Gastric juice is one of the commonly used materials and has significant advantage in the diagnosis of GC.^11^, ^12^, ^15^, ^16^, ^33^ Guo et al explored that some circRNAs in GJ had the potential to be employed as novel indicators for the detection of high‐risk gastric cancer patients.^12^ Shao et al^11^ reported that the levels of miR‐133a in GJ of GC patients were prominently decreased and valuable in the diagnosis of GC. Guo et al disclosed that the abnormality of circ_0065149 expression showed an authentic role in GC oncogenesis, and its existence in exosomes was an biomarker for early screening and prognostic prediction for GC patients.^15^ Virgilio et al indicated that lncRNAs, like H19, UCA1, ABHD11‐AS1, and so forth, were supposed to be the prominent role in GC research, and emerged to be novel potential markers for GC diagnosis.^16^, ^33^ Thus, new biomarker in GJ showed a more distinguished advancement than traditional methods.

In the present study, we first proved the existence of piR‐1245 in GJ. More importantly, traditional tumor biomarkers, such as CEA and CA724, which are simple and easy to screen cancer, have been broadly used for the diagnosis of various tumors, including GC.^5^ However, these markers have low sensitivity or specificity in GC patients. Current experimental results ratified that the significant predictive value of piR‐1245 in human GJ was better than the traditional indicators of GC. These clarified that GJ piR‐1245 had a better distinguishing value to be served as indicator for screening GC.

In current work, we initially found that piRNA‐1245 was overexpressed in GJ from GC patients compared with healthy persons, and for the first time ever, we found that it had good specificity and sensitivity for GC diagnosis. Because gastroscopy could cause an unbearable discomfort in patients which even be life‐threatening. It is difficult for patients to accept as routine examination, especially in developed countries. At present, GJ specimens could be obtained very easily during a physical examination, and it is acceptable to patients.

In conclusion, for the indicator for GC, the use of piRNA‐1245 in GJ has obvious advantages and this research extends our knowledge of non‐invasive biomarker of GC.

## CONFLICT OF INTEREST

None.
